# Outcome of Onlay Mesh Repair for Paraumbilical Hernia: An Experience From a Peripheral Hospital in India

**DOI:** 10.7759/cureus.77564

**Published:** 2025-01-16

**Authors:** Jagannath Hanumanthappa, Sricharan R, Reny Susan N

**Affiliations:** 1 Department of General Surgery, Sri Chamundeshwari Medical College and Hospital, Channapatna, IND; 2 Department of General Surgery, Sri Chamundeshwari Medical College and Hopital, Channapatna, IND

**Keywords:** early complications, onlay mesh repair, paraumbilical hernia, recurrence, seroma

## Abstract

Aim

To know the outcome of onlay mesh repair for paraumbilical hernias that were operated on in a newly established peripheral hospital, mainly emphasizing immediate and delayed postoperative complications.

Methods

This is a retrospective study on 40 patients who were treated by onlay mesh repair at our institution.

Results

Of the 40 subjects who underwent onlay repair, only 10% had developed minor wound-related complications in terms of superficial wound infection and 7.5% developed wound collections (seroma). No instances of recurrence were observed during the study period.

Conclusion

Onlay mesh repair is a relatively easy, quick, and effective procedure with acceptable rates of minor wound complications and also very low/no recurrence rates. It is associated with a low incidence of early wound-related complications. The study did not observe any recurrences within the study period. However, long-term follow-up is needed to determine late recurrence rates. The onlay technique of mesh repair, which is less time-consuming and has acceptable complication rates, provides significant benefits to patients, particularly in peripheral hospitals with limited healthcare resources.

## Introduction

A paraumbilical hernia (PUH) is a defect near the midline close to the umbilicus. PUH can be congenital or acquired. Failure of complete closure of the umbilical ring leads to the development of congenital PUH. The occurrence of umbilical hernia in newborns can be as high as 15% to 16%. Nonetheless, the congenital defect generally closes spontaneously from age three to five. Approximately 90% of PUH diagnosed in adulthood are acquired. The adult acquired hernias account for about 6-14% of all ventral hernias. It is more common in females with a 3:1 female preponderance [1]. In males, it is most commonly seen in the age group of 60 to 70, and in females, it is between 30 and 40 years [1,2]. The causes of PUH in adults include undetected congenital defects that manifest in adulthood, raised intra-abdominal pressure due to various reasons (constipation, chronic liver and kidney diseases), or weakened abdominal wall musculature, especially in females due to repeated pregnancies [3].

The majority of PUH cases remain asymptomatic, with only 3-7% producing symptoms such as pain, swelling, and abdominal discomfort. Pain is the most common symptom (44%). Omentum is the most common content found in the hernial sac but bowel loops are infrequently seen. In 3-5% of cases, PUH can become an abdominal surgical emergency when the contents in the hernia sac become irreducible and strangulated [4].

Annually, approximately 175,000 umbilical hernia repairs are performed in the United States. Recently, an increasing number of individuals with PUH have been seeking treatment, not only in developing countries but also in developed countries like the United Kingdom where surgeries for UH and PUH have increased to 14% from 5% in the last 25 years [3,4]. A similar trend has been observed in Turkey [5]. This increase in incidence is likely due to increased awareness among individuals, the easy availability of surgical expertise even in peripheral centers, and may also be due to cosmetic reasons [6].

Mesh repair remains the preferred treatment for these hernias. The placement of the mesh below the musculoaponeurotic layers (sublay) claimed to be superior to the mesh placement over the musculoaponeurotic layers (onlay) [7,8]. However, in many centers, onlay mesh repairs are being carried out more commonly with comparable outcomes with sublay mesh placement. Studies have shown only minor differences between onlay and sublay techniques in terms of wound-related complications and recurrence rates [9]. Onlay mesh repair is a relatively easy, quick, and straightforward technique with an equally good outcome. Therefore, we decided to conduct a study on the management of PUH in our newly established rural hospital using onlay mesh repair and share our experience with it.

## Materials and methods

Study design and settings

A retrospective observational study was conducted at Sri Chamundeshwari Medical College Hospital at Channapatna, Karnataka, India, from January 2023 to December 2023.

Inclusion criteria

Adults between 18 and 70 years who were diagnosed with PUH and underwent onlay mesh repair were included in this study. Diagnosis of PUH was done by clinical signs and symptoms.

Exclusion criteria

Pediatric patients (less than 18 years), patients with recurrent hernia, and PUH subjects with significant liver disease, ascites, and end-stage renal disease were excluded from the study. Pregnant women with PUH and missing data in the case records were also excluded.

Data collection

All the case files with the diagnosis of PUH were identified and extracted from the medical records department. Subjects fulfilling the study criteria were included and their data were retrieved and documented. This includes patient demography, co-morbid conditions, clinical features, investigations, type of surgery performed, and postoperative complications.

Subjects who underwent surgery for PUH on an elective basis were included in the study. Those who were operated on for emergency reasons like obstructed PUH were excluded. Subjects with co-morbidities like diabetes mellitus, hypertension, obesity, and chronic pulmonary condition were also included in the study, whereas those with ascites due to chronic liver disease and chronic kidney disease were excluded.

The details of the surgical procedure performed, immediate and delayed complications, recurrence, and outcome were noted.

Details of the onlay surgical technique performed

A transverse elliptical infra-umbilical incision was made and the hernial sac was dissected and opened. The contents were reduced to the abdominal cavity. The sac was closed with Vicryl 3.0 and the aponeurotic defect was closed with Prolene number 1.0. The tissue planes all around the defect were dissected quite adequately to fit the size of the mesh. The polypropylene mesh of size 7x5 cm was placed centering the defect. The mesh was anchored all around the rectus sheath with polypropene 2.0 sutures. The vacuum suction drain was placed over the mesh and the subcutaneous tissues and the skin were closed in layers.

The wound was inspected on the third postoperative day and the drain was removed when the drain fluid amount was less than 30 ml. The majority of the patients were discharged on the second or third postoperative day. Skin sutures were removed in the outpatient clinic after 10 days. The patients were followed up in the outpatient clinic after one, three, and six months. They were also advised to come to the outpatient clinic if they had concerning symptoms about the surgical procedure in the follow-up period.

## Results

Fifty cases of PUH who underwent onlay repair were identified and the data were extracted from the medical records room. Out of these 50 cases, only 40 cases met the study criteria and were included in the study. Among them, 24 cases were females (60%) and 16 cases were males (40%). The most common age group for female subjects in our series was 30-40 years (64%), and for male subjects, it was 40-50 years (45%). The most common prevalent symptom was swelling in 22 cases (55%), followed by pain in nine patients (22.5%). The average size of the hernial defect was 3 cm. The most frequent co-morbidity was diabetes mellitus in 12 cases (30%), followed by hypertension in six cases (15%).

The average duration of the surgery was 40 min, with the range being 30-70 min. Postoperative complications were observed in seven cases, with wound discharge being the most common, seen in four cases (10%) and superficial wound collection (seroma) in three cases (7.5%). These wound-related complications were managed with daily dressing, analgesics, and antibiotics. The seromas were treated with daily aspirations until no more collection was evident. The aspirates of seroma were sent for culture and sensitivity, so as the wound discharge from surgical site infections (SSIs). The aspirates of seroma from all three cases and wound swabs from all four cases of SSIs sent for culture resulted in no growth of micro-organisms (Table 1).

**Table 1:** Compilation of results

Parameter	Mean value, range, and SD	
Age	45.7 years (mean) (range 34-70 years), 9.9 years SD	
Sex	Male	13 (32.5%)
	Female	27 (67.5%)
Co-morbidity	Diabetes mellitus type 2	28 (57.5%)
	Hypertension	14 (35%)
	Pulmonary co-morbidities - chronic obstructive pulmonary disease (asthma, chronic bronchitis)	6 (15%)
Duration of hospital stay	3.5 days (mean) (range 2-8 days), 1.8 days (SD)	
Complications	Total number of complications	7 (17.5%)
	Seroma c	3 (7.5%)
	SSI (surgical site infection - All the four cases were non-diabetic)	4 (10%)
Average duration of surgery	40 min (mean) (range 30-70 min), 14 min (SD)	
Average duration of follow-up	4 months (3-6 months)	

Pre-operatively all patients had received intravenous cefotaxime 1 g intravenously the night prior to the day of surgery. The second dose was given at the time of induction of anesthesia and the third dose after 8 h of surgery. For those who developed seroma and SSIs, intravenous cefotaxime was continued twice daily till postoperative day 5. Once the wound-related complications subsided, the patients were discharged with oral cefixime 200 mg for another five days.

The follow-up period ranged from three to six months, with an average follow-up duration of four months. No recurrence was observed in the operated cases during the follow-up period.

## Discussion

The study's findings are very encouraging. This methodology of PUH repair will greatly benefit this population belt as PUH is fairly common in the region though the official prevalence rates are not available.

A meta-analysis conducted by Nguyen MT et al. indicated a higher risk of seroma and SSI in the mesh group compared to the suture repair group. Pooled mesh repairs showed a 2.7% recurrence rate, while suture repairs demonstrated an 8.2% recurrence rate [10].

Another meta-analysis performed by Aslani N et al. had pooled data showing significantly less recurrence when the repair was done with mesh compared to a suture repair (OR 0.09, 95% CI 0.02-0.39). There was no statistically significant difference in the rate of wound complications between the two groups (OR 1.40, 95% CI 0.69-2.84). This indicated a clear benefit of using a mesh repair in reducing recurrence rates without any significant differences in surgical site complication rates between a mesh and a suture repair. The studies may be drawing on skewed data from three randomized trials and only six of 10 observational studies. For definitive conclusions and suggestions, a wider study encompassing the same/similar topic is needed for accurate extrapolation and interpretation of data [11].

Neither of these studies adjusted for the size of the hernia defect, potentially making the hernia defect size a confounding factor. In our study, SSI was found in four cases (10%) and seroma formations were seen in two cases (5%). This is in line with the above studies. However, no recurrences were found in our study.

Dharmendra et al. conducted a study to compare onlay and sublay mesh repairs in 60 patients. In this study, the mean duration of surgery was 46 min in the onlay group. In our study, the mean duration of surgery was 40 min (range 30-70 min). It is very much comparable with both these studies [12].

As per the European Hernia Society and Americas Hernia Society, there is a strong recommendation that mesh be used for the repair of umbilical and epigastric hernias to reduce the recurrence rate. Sutured repair should be considered in shared decision-making and for small hernia defects of less than 1 cm. Thus, mesh repair is advisable in all patients. The plane of mesh placement is an area of debate. In these guidelines, “It is suggested that a flat permanent mesh is placed in the preperitoneal space for open umbilical or epigastric hernia repair”. However, the quality of evidence is poor and the strength of recommendation is weak [12]. Thus, onlay mesh repair is a viable option and the decision is based on the surgeon's preferences/know-how along with the patient's preferences.

An onlay mesh repair could emerge as the preferred operation for small umbilical hernias. It has the potential to reduce the risk of recurrence without significantly increasing the risk of complications like seroma or infection. However, the development of seromas appears to be more likely in onlay repairs than in the sublay technique. In our study, two patients developed seromas. They were treated with oral antibiotics and aspiration drainage at the site of seroma. Antibiotics were used due to the potential risk of mesh infection.

Limitations of this study are as follows: small study population and short duration of follow-up. This study is only a descriptive analysis of the outcome of our method of choice. To assess whether a mesh repair in umbilical hernias reduces the risk of recurrence without significantly increasing surgical site complications, a comparative randomized control trial would be the best option.

## Conclusions

For PUH, onlay mesh repair has the potential to become the surgical treatment of choice. Suture-only repair has become obsolete due to the comparatively better outcome of mesh repairs. Mesh repair being superior in offering tension-free repair for PUHs results in fewer recurrences compared to suture-only repairs. The commonly observed complications like SSIs and seroma formation are slightly more in onlay mesh repairs but that is not statistically significant compared to suture-only repairs. Mesh repair is widely recognized as a successful PUH treatment option. The mesh placement plane with respect to the various musculoaponeurotic layers of the anterior abdominal wall is the only contentious issue surrounding mesh repairs. Despite this, the existing literature leans slightly in favor of sublay repairs due to their lower risk of seroma formation and recurrence compared to onlay repairs. However, onlay repair has some advantages, such as being simple to do and requiring less operating time, which make it a viable option for PUH repair, particularly in peripheral surgical settings where resources may be limited. However, the problem will unquestionably be permanently resolved by multi-institutional randomized controlled studies with a sizable sample size and an extended follow-up period.
